# The mechanism of miR-889 regulates osteogenesis in human bone marrow mesenchymal stem cells

**DOI:** 10.1186/s13018-019-1399-z

**Published:** 2019-11-14

**Authors:** Gang Xu, Zheng Ding, Hui-feng Shi

**Affiliations:** 1 Department of Orthopedics, Xuzhou Medical University affiliated Hospital of Lianyungang, Lianyungang, 222061 Jiangsu Province China; 2 Department of Orthopedics, TongRen Hospital, Shanghai Jiaotong University School of Medicine, 1111 Xianxia road, Shanghai, 200336 China

**Keywords:** miR-889, Osteogenic differentiation, Bone marrow mesenchymal stem cells

## Abstract

**Background:**

Bone marrow mesenchymal stem cells (BMMSCs) can be used for bone regeneration in the specified condition. Osteogenic differentiation of BMMSCs is controlled by microRNAs (miRNAs) and other factors. This study was aimed to identify the role and mechanism of miR-889 in regulating the osteogenic differentiation of BMMSCs.

**Methods:**

Osteoporosis patients and normal control bone tissues were collected and used PCR techniques to identify the change of miR-889 and WNT7A. Moreover, the dynamic change of miR-889 and WNT7A during osteogenic differentiation of BMMSCs was also measured. Bioinformatic analysis was performed to identify the target genes and potential pathways of miR-889. Then, we constructed miR-889 mimic and inhibitor, ALP staining, ARS, osteoblastic-related protein, and Wnt β-catenin signaling pathway-related protein were also measured. WNT7A siRNA was also used to verify the function of miR-889.

**Results:**

In the present study, we showed that miR-889 expression was upregulated in osteoporosis patients than healthy control. However, the miR-889 expression was downregulated during osteogenic differentiation. Bioinformatics analysis found that miR-889 targets 666 genes and mainly through Wnt β-catenin signaling pathway. Administrated miR-889 mimic, the ALP activity, and calcium deposition were decreased than the control group, while miR-889 inhibitor shown the opposite trend. And miR-889 could bind the 3′UTR of WNT7A. We further used WNT7A siRNA to explore the function of miR-889, and the results revealed that co-cultured with miR-889 inhibitor and WNT7A siRNA was associated with a reduction of ALP activity and calcium deposition and osteoblastic-related proteins than miR-889 inhibitor alone.

**Conclusion:**

Our results revealed that miR-889 plays a negative role in inducing osteogenic differentiation of BMSCs through Wnt β-catenin signaling pathway.

## Background

Bone marrow mesenchymal stem cells (BMMSCs) have been reported to be a population of self-renewing and multidirectional differentiation cells [1, 2]. BMMSCs have the potential to differentiate into osteoblasts, chondrocytes, and adipocytes [3]. The decreased ability of osteogenic potential of osteoblasts from BMMSCs is the major risk of osteoporosis [4]. However, the mechanism about the molecular and potential pathways remains unclear [5, 6].

MicroRNAs (miRNAs), which are a group of endogenous small non-coding RNAs, play important roles in post-transcriptional regulation of osteogenic differentiation of BMMSCs [7, 8]. MiRNAs negatively regulate the expression of their target genes post-transcriptionally, by directly binding to a partially complementary sequence in the 3′ untranslated region (3′-UTR) [9, 10]. A variety of miRNAs have been shown to be regulated during osteogenic differentiation of BMMSCs. For example, miR-206 inhibits osteogenic differentiation of BMMSCs by targeting glutaminase [11]. Additionally, miR-214 could suppress the osteoblast differentiation by modulating JNK and p38 signaling pathway [12].

Previously, Han et al. [13] revealed that miR-889 inhibits non-small cell lung cancer progression by targeting Krueppel-like factor 9 (KLF9). Ge et al. found that hsa-miR-889 promotes the proliferation of osteosarcoma through inhibiting myeloid cell nuclear differentiation antigen expression [14]. Previously, we found that miR-889 was increased in osteoporosis patients than that of normal patients. However, the function of miR-889 during osteoblast differentiation is poorly understood. Wnt/β-catenin signaling pathway is critically important in regulation osteoblastic differentiation of BMMSCs [15]. WNT7A binds to the receptor (low-density lipoprotein receptor (LRP) 5/6 and frizzled) and thus lead β-catenin degradation [16].

In this study, the expression and correlation of miR-889 and WNT7A was determined by osteoporosis patients and healthy controls. Meanwhile, we used bioinformatics analysis to identify the targeting sites and potential pathways of miR-889. Furthermore, both loss-of-function and gain-of-function were performed to analyze the role of miR-889 in osteogenic differentiation.

## Materials and methods

### Patients and sample collection

This study was approved by the Institutional Ethical Committee of TongRen Hospital, Shanghai Jiaotong University School of Medicine. Written informed consent was signed by all included patients. Inclusion criteria were (1) bone mineral density (BMD) of at least 2.5 standard deviation (SD) below the peak mean BMD of healthy young women (− 2.5 T-score), (2) initial diagnosis of osteoporosis (OP) and receive no drug or hormone treatment, and (3) no thyroid disease. Finally, we included 6 women with OP and 5 healthy controls for further analyses of fresh femoral neck trabecular bone from osteoporotic undergoing hip replacement due to either osteoporotic fracture (OP group, *n* = 6) or osteoarthritis in the absence of OP (control group, *n* = 5).

### Cell culture and differentiation

Human BMMSCs were obtained from Cyagen company (Cyagen Biosciences; Guangzhou, China) and cultured in the α-MEM containing 10% fetal bovine serum (ThermoFisher, USA). For osteogenic differentiation, the BMSCs were cultured in an induction medium containing 50 mM ascorbic acid, 10 mM sodium b-glycerophosphate, and 10 nM dexamethasone according to previous report [17]. Cells were harvested at the indicated times for mRNA and miRNA extraction. Negative control (NC), miR-889 mimic, miR-889 inhibitor, and WNT7A siRNA were obtained from GenePharm, Tech, (Shanghai, China). The sequences of miR-889 mimics were the primary chain, 5′-ACACTCCAGCTGGGAATGGCTGTCCGTAGT-3′, and passenger chain, 5′-TGGTGTCGTGGAGTCG-3′. The concentrations of miR-889 mimics and miR-889 inhibitor used were 100 nmol/L.

### Quantitative real-time polymerase chain reaction (qRT-PCR) analysis

Samples were harvested from OP patients and healthy controls. HBMSCs that used for osteogenic induction were collected after 21 days of intervention. After washing by PBS for three times and smashed. Then, total RNA is extracted by TRIzol reagent (Invitrogen, USA) according to the manufacturer’s instructions. Real-time PCR was performed using the miScript SYBR Green PCR kit (Qiagen) for miRNA expression analysis and a standard SYBR Green PCR kit (Takara, Tokyo, Japan). The relative changes of transcripts of interest were analyzed according to the 2^-△△ct^ method with GAPDH and U6 being housekeeper genes. Primer sequences are available in Table 1.

**Table 1:** The gene primer used in this study

Gene names	Sequence
WNT7A	Forward 5′-TGG ATG CCC GGG AGA TC-3′
	Reverse 5′-CCG ACC CGC CTC GTT ATT-3′
miR-889	Forward 5′ACACTCCAGCTGGGAATGGCTGTCCGTAGT 3′
	Reverse 5′TGGTGTCGTGGAGTCG 3′
U6	Forward 5′CTCGCTTCGGCAGCACATATACT3′
	Reverse 5′ACGCTTCACGAATTTGCGTGTC3′
GADPH	Forward 5′AAGGTGAAGGTCGGAGTCA3′
	Reverse 5′GGAAGATGGTGATGGGATTT3′

### Bioinformatics analysis

First, a Venn diagram was produced to show the potential common target genes of miR-889 in Targetscan (http://www.targetscan.org/vert_72/), miRDB (http://mirdb.org/), and miRanda (http://www.microrna.org/microrna/home.do) databases. Then, DAVID Bioinformatics Resources 6.8 (https://david.ncifcrf.gov/) was used to verify the Gene Ontology (GO) function of these target genes. Kyoto Encyclopedia of Genes and Genomes (KEGG) pathway analysis was also performed to identify the potential pathway of miR-889 that involved. Binding sites of miR-889 and WNT7A were performed by Targetscan databases.

### Western blot analysis

Total proteins are extracted by 1 ml RIPA mixed with PMSF. The isolated protein concentration was determined using BCA Protein Assay Kit (LEAGENE, Beijing, China). Equally amount of protein (20 μg) was separated on SDS-PAGE, electrotransferred to polyvinylidene difluoride (PVDF) membranes. Following blocking within Tris-buffered saline and Tween 20 (TBST) containing 5% skim milk, membranes were incubated with primary antibodies against ALP, BMP-2, RUNX2, OPN, and OCN (1:1000) all from Santa Cruz (USA) overnight at 4 °C. Then, membranes were washed by TBST for 5 min for three times. Samples were incubated with HRP-conjugated second antibodies (1:5000; Santa Cruz, CA, USA) for 2 h to detect immunoreactive bands. Blotted bands were visualized with ECL solution (Boster, Wuhan, China) and exposed to films.

### Alkaline phosphatase staining (ALP) staining and alizarin red staining (ARS)

After osteogenic induction for at least 7 days, each plate was washed by PBS for three times and then fixed by 4% paraformaldehyde for 15 min. Then, each plate was coated by nitro-blue tetrazolium chloride (BCIP)/5-bromo-4-chloro-3′-indolyphosphate p-toluidine salt (NBT) solution for 5 min. Then, plates were washed by water to stop the reaction.

ARS was performed to detect calcium deposition in each group. After osteogenic induction for 14 days, each well was fixed by 4% paraformaldehyde for 15 min. Then, we added Alizarin Red solution (0.1%, Ph = 6.8) for 45 min. Stained cells were then photographed after used water to stop the reaction.

### Immunofluorescence staining

To identify the β-catenin activity in response to miRNA treatments, immunofluorescence staining was performed for NC, miR-889 inhibitor, and miR-889 inhibitor+WNT7Ai groups. In briefly, hBMSCs were transfected with NC, miR-889 inhibitor, and miR-889 inhibitor+WNT7Ai, respectively. After osteogenic induction for 7 days, differentiated hBMSCs were harvested and used for immunofluorescence staining. HBMSCs were fixed by 4% paraformaldehyde for 30 min and then washed by PBS foe three times. After blocking with 1% BSA for 30 min, hBMSCs were incubated with primary antibody for β-catenin at 37 °C for 2 h; then, second antibody was added for fluorescence microscope (Olympus, Japan).

### Statistical analysis

Outcomes are shown as the mean ± standard deviation (SD). Correlation between WNT7A and miR-889 was calculated by Pearson correlation coefficient. Statistical analysis was performed with *t* test between two groups and analysis of variance (ANOVA) if more than the two groups compared using SPSS statistics 21.0 (IBM Corp., Armonk, NY, USA). *P* values < 0.05 were considered as statistically significant.

## Results

### miR-889 is upregulated in OP patient

**Fig. 1:**
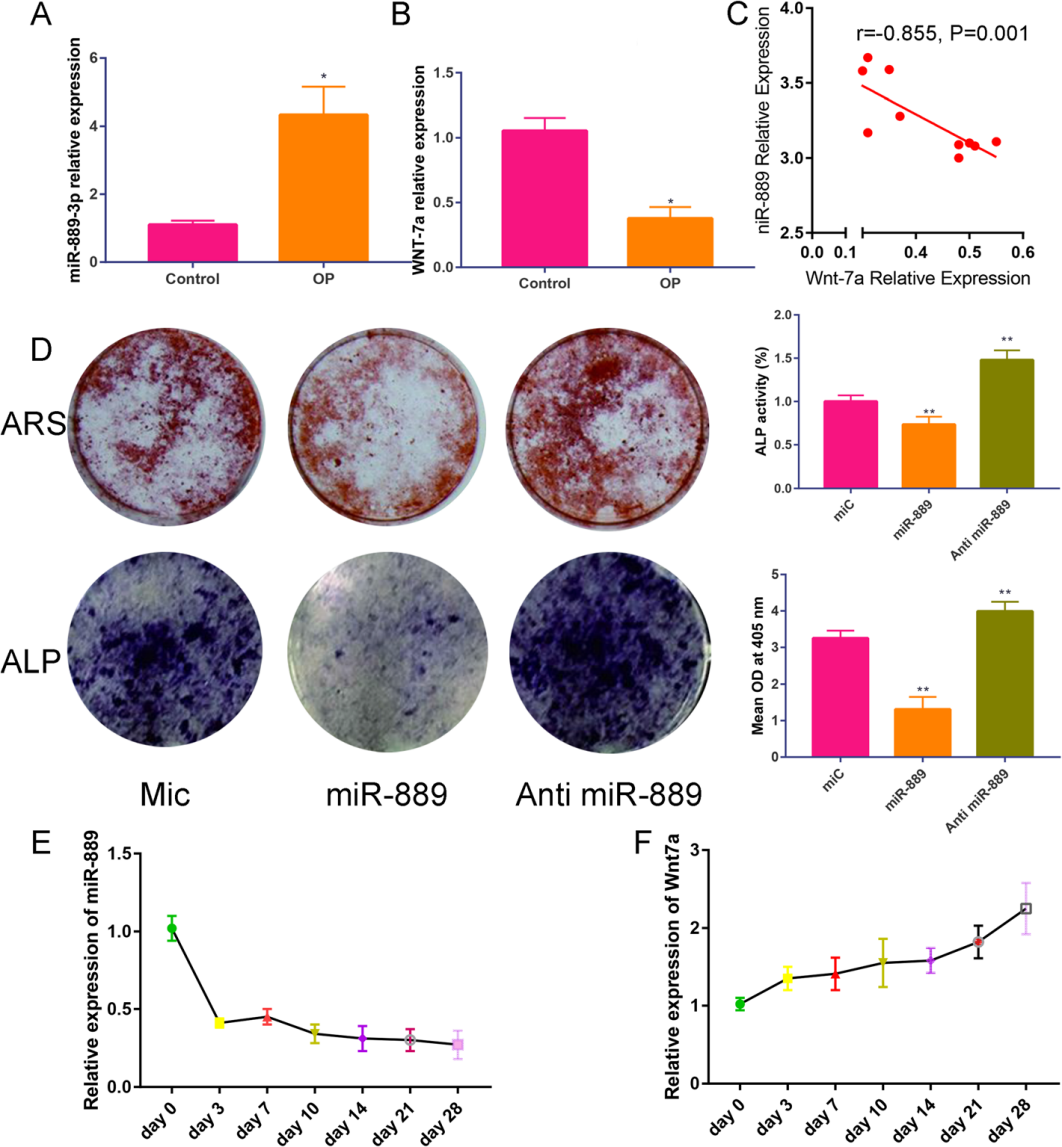
**a** Relative expression of miR-889 in osteoporosis patients and healthy control. **b** Relative expression of WNT7A in osteoporosis patients and healthy control. **c** Correlation of miR-889 and WNT7A. **d** ALP staining and ARS in Mic, miR-889, and anti-miR-889. **e** Relative expression of miR-889 during osteogenic differentiation of BMMSCs (from day 0 to 28). **f** Relative expression of WNT7A during osteogenic differentiation of BMMSCs. *P < 0.05 compared with the healthy control group. **P < 0.01 compared with the Mic group

As illustrated in Fig. 1, we found that the expression of miR-889 is increased in OP patients than the healthy control group. However, the WNT7A was decreased in OP patients than the healthy control group. Moreover, we found that the relative expression of WNT7A and miR-889 have a negative correlation (*r* = − 0.855, *P* = 0.001).

Figure 1b revealed the ALP and ARS staining; in accordance with general observation, ALP staining and red calcium nodules were decreased in miR-889 group, while increased in anti-miR-889 with statistically significant.

We further compared the miR-889 and WNT7A expression during osteogenic differentiation of hBMMSCs. The results are presented in Fig. 1c. Osteogenic-induced medium group was associated with an increased expression of miR-889, while with a reduction expression of WNT7A than the control group. Moreover, ALP staining and ARS results were presented in Fig. 1d, and we found that miR-889 could significantly decrease the ALP activity and calcium deposit than the control group while miR-889 inhibitor could significantly increase ALP activity and calcium deposit than the control group. As illustrated in As the time of osteogenic induction time prolong, the miR-889 expression was decreased (Fig. 1e), while WNT7A relative expression increased (Fig. 1f).

### Bioinformatics analysis

**Fig. 2:**
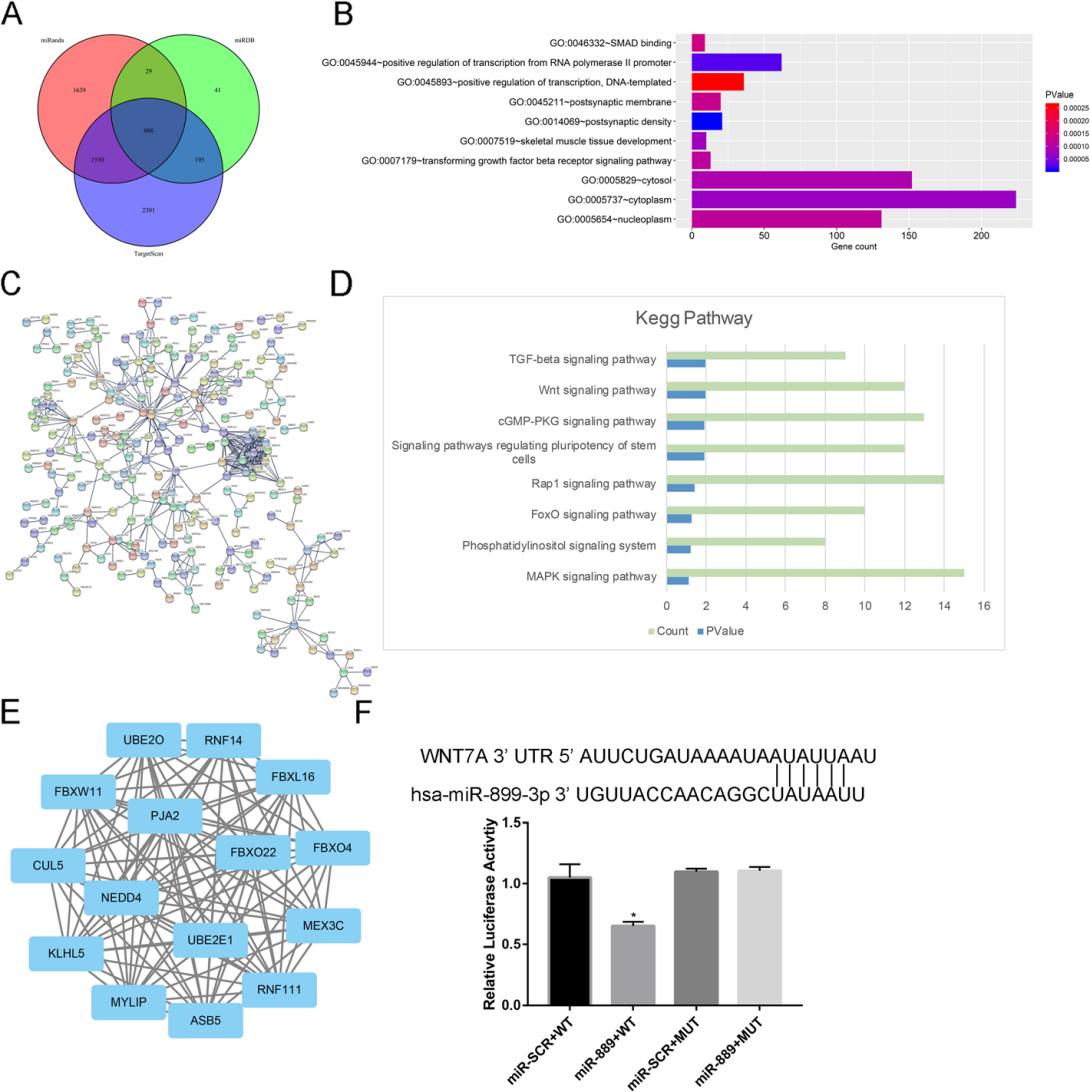
**a** Venn diagram of the target genes of miR-889 in Targetscan, miRDB, and miRanda databases, **b** Gene Ontology of the target genes. **c** Protein-protein interaction of the target genes. **d** Kegg pathway of the target genes. **e** MCODE results from protein-protein interaction. **f** Binding sites of the miR-889 and WNT7A

Firstly, we used three target gene prediction websites (miRanda, miRDB, and Targetscan) to verify the target genes of miR-889. Finally, we identify 666 target genes through Venn diagram (Fig. 2a). As listed in Fig. 2b, these 666 target genes gathered in the following Gene Ontology: SMAD binding, positive regulation of transcription from RNA polymerase II promoter, positive regulation of transcription, DNA templated, and postsynaptic membrane. Figure 2c shown the interaction between the target genes, and we further used MCODE model to identify the hub genes. Hub genes of the PPI were shown in Fig. 2e. The hub gene of the PPI was the FBXO22 gene. Figure 2d revealed that 8 potential pathways were enriched: TGF-beta signaling pathway, Wnt signaling pathway, cGMP-PKG signaling pathway, Rap1 signaling pathway, FoxO signaling pathway, phosphatidylinositol signaling pathway, and MAPK signaling pathway. Figure 2f revealed the binding sites of miR-889 and WNT7A.

### Overexpression and inhibition of miR-889 can affect osteoblast differentiation

**Fig. 3:**
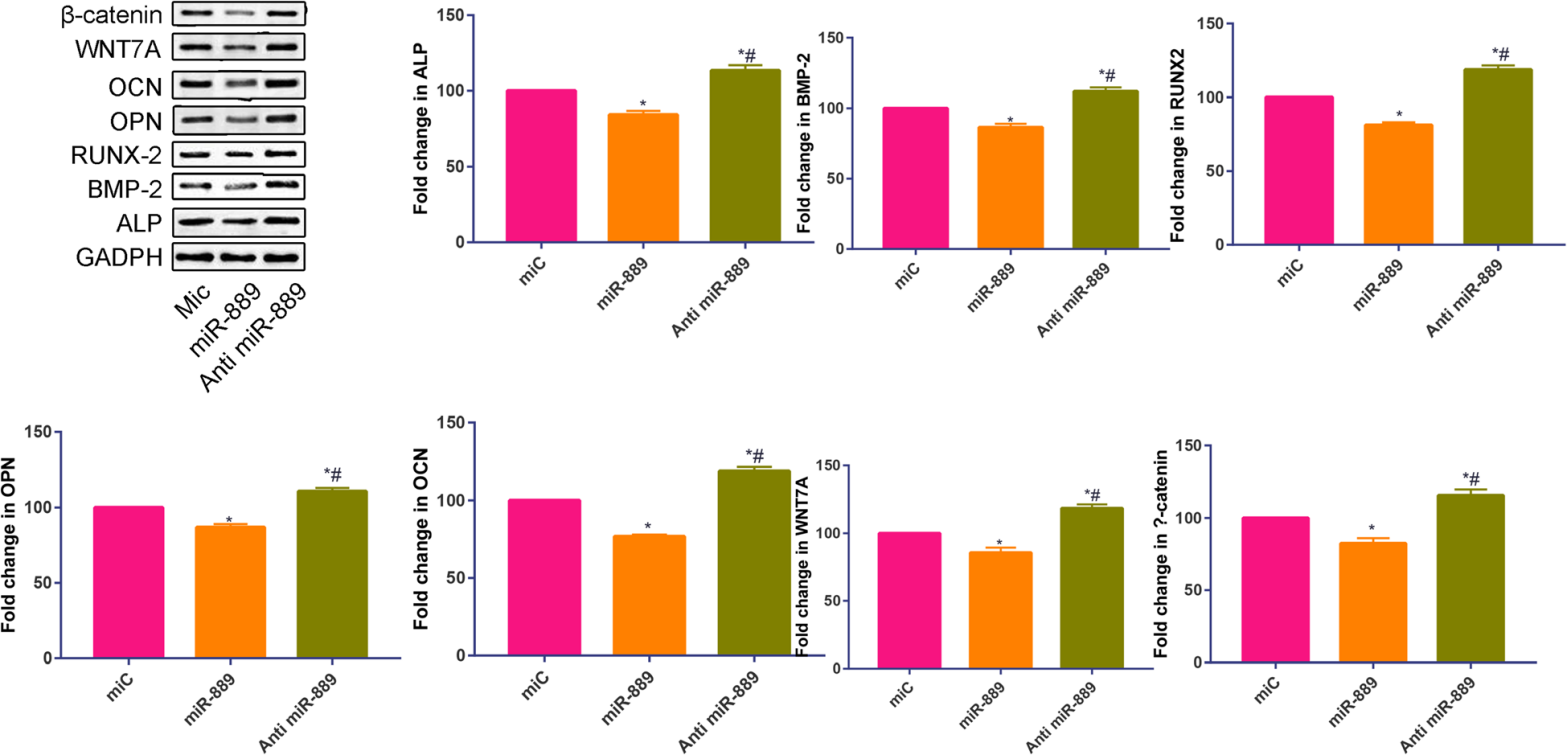
Relative protein expression of ALP, BMP-2, RUNX2, OPN, OCN, WNT7A, and β-catenin in Mic, miR-889, and anti-miR-889 groups. *P < 0.05 compared with Mic group. #*P* < 0.05 compared with miR-889 group

To further determine the role of miR-889 in osteoblastic differentiation, the BMSCs were infected with negative control (Mic) or transfected with mimic (miR-889) or inhibitors (Anti miR-889) individually. Results were shown in Fig. 3. We found that compared with negative control, miR-889 mimic was associated with a decrease of the osteoblastic markers including ALP, BMP-2, RUNX-2, OPN, and OCN. And, when administrated with anti-miR-889, osteoblastic markers (ALP, BMP-2, RUNX-2, OPN, and OCN) were increased than the negative control and miR-889 mimic. Moreover, we measured Wnt-β catenin signaling pathway-related protein (Wnt7a and β-catenin). We found that miR-889 mimic could significantly reduce the Wnt7a and β-catenin expression. And anti-miR-889 significantly increased Wnt7a and β-catenin expression.

### miR-889 regulates osteoblast differentiation by targeting WNT7A and through Wnt-β catenin signaling pathway

**Fig. 4:**
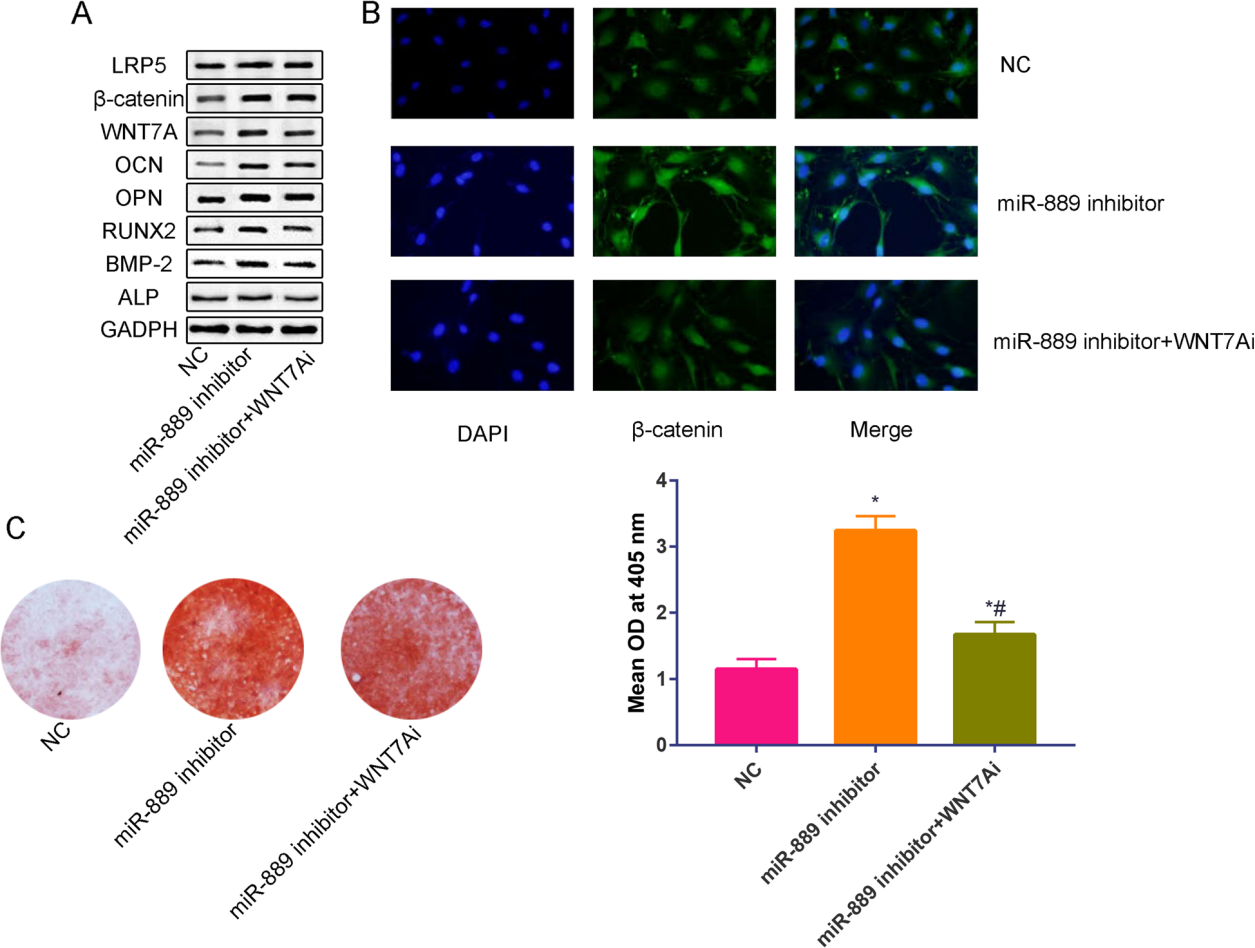
**a** Relative expression of ALP, BMP-2, RUNX2, OPN, OCN, WNT7A, β-catenin, and LRP5 in NC, miR-889 inhibitor, and miR-889 inhibitor+WNT7Ai groups. **b** immunofluorescence results of β-catenin expression in NC, miR-889 inhibitor, and miR-889 inhibitor+WNT7Ai. **c** ARS results in NC, miR-889 inhibitor, and miR-889 inhibitor+WNT7Ai. **d** Quantitative analysis of the calcium deposition in NC, miR-889 inhibitor, and miR-889 inhibitor+WNT7Ai. *P < 0.05 compared with Mic group. #*P* < 0.05 compared with miR-889 group

We further used negative control, miR-889 inhibitor, and miR-889 inhibitor combined with WNT7A inhibitor to further analyze the miR-889/Wnt-β catenin axis in inhibiting osteogenesis of hBMSCs. Compared with NC, miR-889 inhibitor decreased the protein level of osteogenic markers (ALP, BMP-2, RUNX-2, OPN, OCN, Fig. 4a) and Wnt-β catenin signaling pathway-related proteins (WNT7A, β-catenin, and LRP5, Fig. 4a). In contrast, when co-cultured miR-889 inhibitor and WNT7Ai, the protein level of osteogenic markers (ALP, BMP-2, RUNX-2, OPN, OCN) was decreased than miR-889 inhibitor alone. Furthermore, we used immunofluorescence to identify the expression of β-catenin in negative control, miR-889 inhibitor and miR-889 inhibitor+WNT7Ai groups (Fig. 4b). Results found that miR-889 inhibitor significantly increased the β-catenin expression level, while co-cultured with WNT7A inhibitor, the β-catenin expression level decreased to some extent. ARS staining was used to further identify miR-889/Wnt-β catenin signaling pathway involved in regulating the osteogenic differentiation of hBMMSCs (Fig. 4c).

## Discussion

**Fig. 5:**
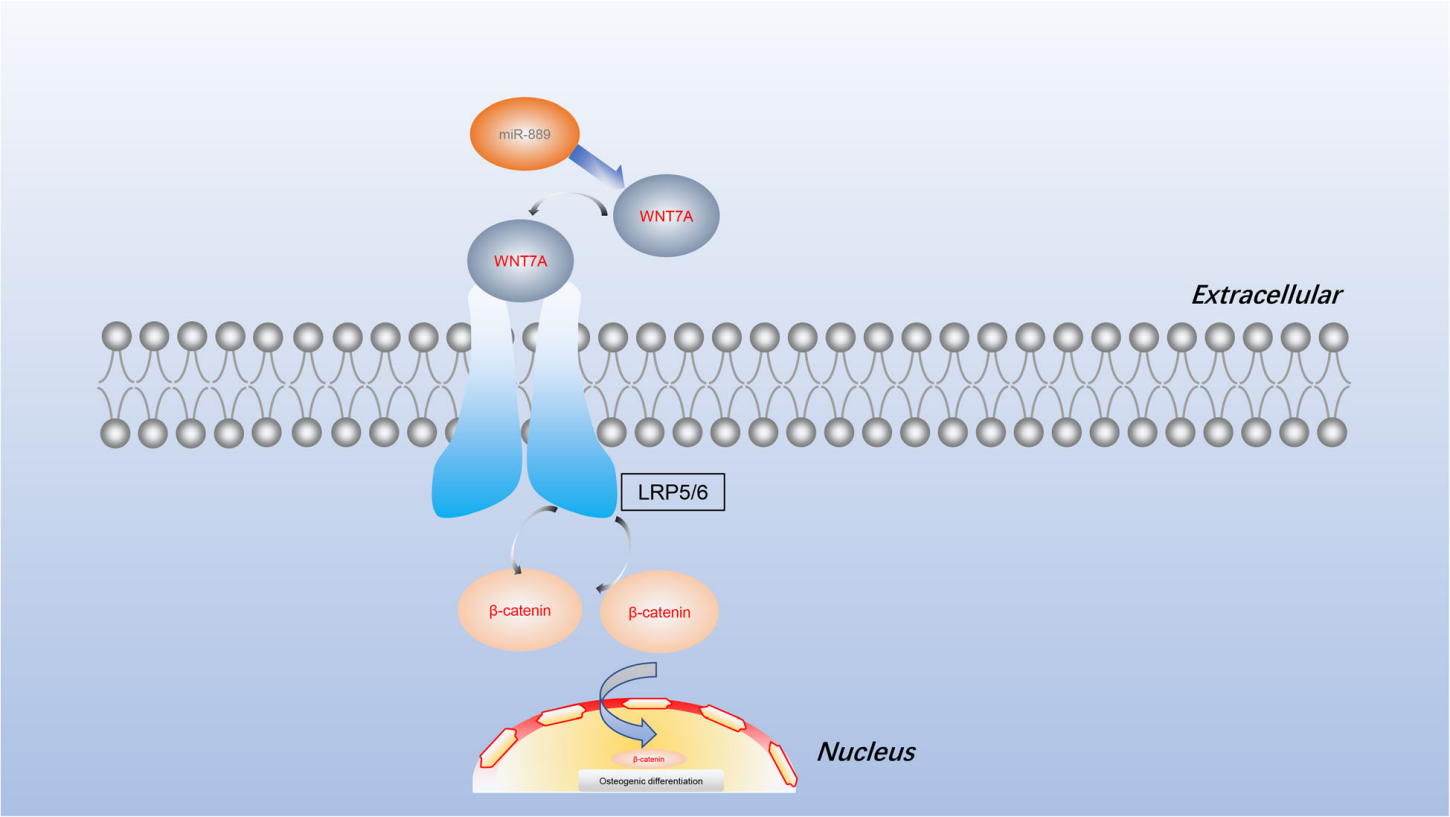
Schematic diagram of miR-889 regulates osteoblastic differentiation of hBMMSCs by targeting WNT7A and through Wnt β-catenin signaling pathway

Our results revealed that miR-889 negatively regulate WNT7A-mediated Wnt-β catenin signaling pathway and thus inhibit osteogenic differentiation of hBMMSCs. First, we observed miR-889 was upregulated in OP patients and has a negative correlation with WNT7A. Second, miR-889 was downregulated and WNT7A was upregulated during osteogenic induction of hBMMSCs. Third, bioinformatics analysis identifies the miR-889 targeting WNT7A and possibly through Wnt β-catenin signaling pathway. Fourth, inhibition of miR-889 enhances the osteogenic differentiation in vitro, while the mimic of miR-889 had the opposite effects (Fig. 5).

MiR-889, as a novel miRNA, was not reported in osteogenic differentiation of hBMMSCs. Several studies reported that the role and function of miR-889 in colorectal cancer [18], osteosarcoma [14], and lung cancer [13]. In this research, we firstly used a clinical sample to identify the relative expression of miR-889 and WNT7A. We found that miR-889 was upregulated in OP patients, while WNT7A had an opposite tendency. In contrast, miR-889 was downregulated during osteogenic differentiation in hBMMSCs. Thus, we speculated that miR-889 has a negative role in regulating osteogenic differentiation of hBMMSCs. Overexpression and inhibition approaches were used to identify the function of miR-889 in osteoblast differentiation of hBMMSCs. ALP and ARS were used to identify the early and late stage of osteogenic induction of hBMMSCs, respectively. We found that, compared with NC group, miR-889 mimic is associated with a reduction of ALP activity and calcium deposition.

We performed bioinformatics analysis to identify the target genes of miR-889. Venn diagram found that a total of 666 potential genes intersect in Targetscan, miRDB, and miRanda databases. DAVID Bioinformatics Resources 6.8 is used to reveal the target genes’ function. And FBXO22 gene was identified as the hub gene. Kegg pathway result found that Wnt β-catenin signaling pathway possibly involved in the miR-889 inhibiting osteogenic differentiation of hBMMSCs. MiR-889 have a total of 6 binding sites with WNT7A.

Osteoblastic-related proteins (ALP, BMP-2, RUNX2, OPN, and OCN) were used to identify the osteogenic capability [19]. Compared with NC, miR-889 mimic was associated with a reduction of these osteoblastic-related proteins. However, when administrated miR-889 inhibitor, these osteoblastic-related proteins were upregulated. These results further confirmed that miR-889 has a negative role in regulating osteoblastic induction of hBMMSCs. Dozens of miRNAs have potential to regulating Wnt β-catenin signaling pathway. Long et al. [20] revealed that miR-381 modulates hBMMSC osteogenesis via suppressing Wnt signaling pathway during atrophic nonunion development. Li et al. [21] found that miR-23a inhibits osteogenic differentiation of hBMMSCs by targeting LRP5 (Wnt receptor). When administrated with miR-889 mimic, we found that WNT7A and β-catenin was reduced; however, miR-889 inhibitor has an opposite tendency. These results indicated that WNT7A and β-catenin were the downstream genes of miR-889.

To further identify the role of WNT7A in regulating osteoblastic induction of hBMSCs, we used WNT7A siRNA to verify the function of WNT7A. Co-cultured with miR-889 inhibitor and WNT7Ai were associated with a reduction of the calcium deposition than miR-889 alone and also increased than NC group. Zheng et al. [22] found that disturbing the Wnt-β catenin signaling pathway could impair the osteogenesis of hBMMSCs. Our results were in accordance with previous findings. Wnt β-catenin signaling pathway could also promote the human amniotic epithelial cells into osteoblasts [23]. When administrated with miR-889 inhibitor, we found that β-catenin nuclear translocation increased and the fluorescence intensity were also increased.

In conclusion, we found that miR-889 inhibit the osteogenic differentiation of hBMMSCs through WNT7A-mediated Wnt β-catenin signaling pathway. Further study should be focused on the role of miR-889 in regulating osteogenesis in vivo.

## Data Availability

The datasets used and analyzed during the current study are available from the corresponding author on reasonable request.

## Abbreviations

BMMSCs: Bone marrow mesenchymal stem cells
miRNAs: MicroRNAs
3′-UTR: 3′ untranslated region
KLF9: Krueppel-like factor 9
LRP5: Low-density lipoprotein receptor
BMD: Bone mineral density
SD: Standard deviation
OP: Osteoporosis
NC: Negative control
GO: Gene Ontology
KEGG: Kyoto Encyclopedia of Genes and Genomes
PVDF: Polyvinylidene difluoride
ARS: Alizarin Red staining
\ANOVA: Analysis of variance
ALP: Alkaline phosphatase staining
